# Supplementary material for: Cloning and Functional Analysis of NoMYB60 Gene Involved in Flavonoid Biosynthesis in Watercress (Nasturtium officinale R. Br.)
Source: Genes (Basel). 2022 Nov 14;13(11):2109. doi: 10.3390/genes13112109 (PMC9690578; doi:10.3390/genes13112109)
Supplement: Supplementary file 1 [file genes-13-02109-s001.zip › genes-1965397-supplementary.pdf]

Table S1 Primers used for cloning and vector construction

Primer	Sequence (5'-3')
Cloning	
NoMYB60-F	ATGGGTAGGCCTCCATGCTG
NoMYB60-R	TTAGAGCATATTAGAGAGCT
Overexpression	
NoMYB60-F-NdeI	TCTTCACTGTTGATACATATGATGGGTAGGCCTCCATGCTG
NoMYB60-R-BamHI	GCCCTTGCTCACCATGGATCCGAGCATATTAGAGAGCT
Yeast two hybrid	
BD-NoMYB60-F	ATGGCCATGGAGGCCGAATTCATGGGTAGGCCTCCATGCTG
BD-NoMYB60-R	CCGCTGCAGGTCGACGGATCCGAGCATATTAGAGAGCT
BD-NoMYB60-1-F	ATGGCCATGGAGGCCGAATTCATGGGTAGGCCTCCATGCTG
BD-NoMYB60-1-R	CCGCTGCAGGTCGACGGATCCGTTATCATTCTCTGAAATCT
AD-NoBEH1-F	GCCATGGAGGCCAGTGAATTCATGGCGGCCGGAGGAGGTGG
AD-NoBEH1-R	CAGCTCGAGCTCGATGGATCCGCATCGTATTTTAGTGC
AD-NoBEH2-F	GCCATGGAGGCCAGTGAATTCATGGCCGCCGGAGGTGGATG
AD-NoBEH2-R	CAGCTCGAGCTCGATGGATCCGCATCGGGTATTAGTGC
BiFC	
YC-NoMYB60-F	GGACCTGCTTTCTAGAATGGGTAGGCCTCCATGCTG
YC-NoMYB60-R	CGGGTACCGGATCCGAGCATATTAGAGAGCT
YN-NoBEH1-F	TCTCGAGGGATCCATGGCGGCCGGAGGAGGTGG
YN-NoBEH1-R	ATGGATCTTCTAGAGCATCGTATTTTAGTGC
YN-NoBEH2-F	TCTCGAGGGATCCATGGCCGCCGGAGGTGGATG
YN-NoBEH2-R	ATGGATCTTCTAGAGCATCGGGTATTAGTGC
qRT-PCR	
qNoPAL-F	GACCGTGAGCAAGTCTACACA
qNoPAL-R	GCGTTCTTCTCACTCTCACCA
qNoC4H-F	TACGATATCCCTGCGGAAAGC
qNoC4H-R	GGACAGCTTCTACGTCCAACA
qNo4CL-F	AGGTGCTGCTCCTCTAGGTAA
qNo4CL-R	ATCCACGATCTTCATCTCGGC

qNoCHs1-F	GACAATGCCAACATGTGTGCT
qNoCHs1-R	TGATCTTGGACTTGGGCTGAC
qNoF3H-F	TTAGCAACGAAATCCCGGTCA
qNoF3H-R	CCTGGAAGATGCCCCAATTCT
qNoDFR-F	CGTGGGATTATGCCAAGGAGA
qNoDFR-R	ATGTGGCTTGTTCCGAGTGAT
qNoANS-F	TGCAGCTTTTCTATGAGGGCA
qNoANS-R	TCTCCTTATTCACCAACCCGC
qNoUFGT-F	CCGACGTTCCCTTCTTCTTGA
qNoUFGT-R	CACGATGTTGAAGCAAACGGT
qNoActin-F	TCCAACGCTAGCTGCACCAC
qNoActin-R	AGCAGCTCTTCCACCTCTCCA
qAtActin-F	TTGACAATTGATGCAAACAAT
qAtActin-R	CCATTGCTTAATTCCACGGAC

Table S2 Sequences of oligonucleotides used for VIGS

Gene Name	Sequence (5'-3')
NoMYB60	TTGACATTTATTGAGAAATGGCTTTTGGAGGAAACAAGTA
	TACTTGTTTCCTCCAAAAGCCATTTCTCAATAAATGTCAA

Table S3 Accession number of genes in NCBI

Gene name	Accession
AtMYB60	NP_172358.1
OsMYB306-1	XP_015616542.1
OsMYB306-2	XP_015619015.1
ZmMYB306	XP_008652703.1
TaMYB60-like	XP_044377782.1
SlMYB306	XP_004236011.1
SlMYB306-like	NP_001304303.1
DcMYB306	XP_017257203.1
DcMYB306-like	XP_017238412.1
BcMYB60	ADQ92843.1
